# An Atlas of Genetic Correlations Between Thyroid Hormone Levels and Human Health‐Related Traits

**DOI:** 10.1002/hsr2.71092

**Published:** 2025-07-21

**Authors:** James L. Li, Yijia Sun

**Affiliations:** ^1^ Department of Public Health Sciences University of Chicago Chicago Illinois USA; ^2^ Interdisciplinary Scientist Training Program University of Chicago Chicago Illinois USA

**Keywords:** correlation, endocrinology, genetics, genomics, GWAS, thyroid

## 1 Introduction

Thyroid hormones are essential for normal human development and the regulation of metabolism [1, 2]. These hormones, including thyroid stimulating hormone (TSH), free triiodothyronine (FT3), total triiodothyronine (TT3), and free thyroxine (FT4), are routinely used in clinical practice as markers for screening and diagnosing thyroid dysfunction [3]; TSH serves as the primary screening test for patients with suspected thyroid disorders, while thyroid tests that measure FT3, TT3, and FT4 levels are used to diagnose thyroid diseases [3, 4]. Previous epidemiological studies have shown that thyroid hormone levels are associated with many diseases and health conditions in humans, including cardiovascular diseases [5, 6], psychiatric disorders [7, 8], sleep duration [9], and cancers at various body sites [10, 11, 12]. However, the etiology underlying these associations is not fully understood.

Genetic correlation has emerged as a metric to quantify the degree of similarity between two traits based on shared genetic variations identified in genome‐wide association studies (GWAS) and has enhanced our understanding of the etiology underlying many complex traits [13, 14, 15]. Though large‐scale GWAS have helped in mapping the genetic bases of many human diseases and health‐related traits [16, 17, 18, 19, 20], GWAS of thyroid hormones have been historically limited by lower sample sizes [21, 22] with the exception of a recent GWAS involving up to 271,040 individuals of European ancestry that identified 413 independent genetic variants associated with levels of several thyroid hormones [23]. Thus far, the shared genetic bases between thyroid hormones and other health‐related traits and diseases has not been fully explored. In this study, we aimed to systematically compute the genetic correlation between each of four thyroid hormones (TSH, TT3, FT3, FT4) and numerous health‐related traits such as aging, cancer, gastrointestinal disease, psychiatric‐neurologic disorders, and blood‐related, cardiometabolic, immune‐related, and anthropometric traits.

## 2 Methods

### 2.1 Obtaining GWAS Summary Statistics for Thyroid Hormones and Other Health‐Related Traits/Diseases

GWAS summary statistics for TSH, TT3, FT3, and FT4 were obtained from Sterenborg et al. [23] and were based on 271,040, 15,829, 59,061, and 119,120 individuals, respectively. GWAS summary statistics for 72 health‐related traits and diseases that were not directly related to thyroid function or thyroid‐related disease [18, 24, 25, 26, 27, 28, 29, 30, 31, 32, 33, 34, 35, 36, 37, 38, 39, 40] were obtained and processed as described in Barbeira et al. [41] from 85 previously conducted GWAS, and involved individuals across 18 consortia listed in Supporting Information S1: Table 1. We generally classified these traits/diseases into the following 10 categories: aging, cancer, gastrointestinal disease, psychiatric‐neurological disease, skeletal disease, and anthropometric, blood‐related, cardiometabolic, immune‐related, and morphological traits (Supporting Information S1: Table 1). We selected these 72 traits for several reasons. First, these traits are all complex traits that encompass a wide range of physiological and disease‐related states, thus allowing for a comprehensive evaluation of genetic correlations between thyroid hormones and human health and disease. In addition, previous literature has provided evidence suggesting that each of these traits and diseases has some genetic basis, with nearly all these traits having reported genome‐wide significant loci [18, 24, 25, 26, 27, 28, 29, 30, 31, 32, 33, 34, 35, 36, 37, 38, 39, 40]. Lastly, these GWAS were harmonized and processed using identical protocols including variant imputation and standardization, and therefore had consistent imputation quality across traits [25, 41]; this consistency in post‐GWAS harmonization enabled standardized comparison across traits in the genetic correlation analyses conducted in this study. All original studies involved in these GWAS received ethical approval, and participants provided informed consent.

### 2.2 Computing Genetic Correlations Between Thyroid Hormones and Other Health‐Related Traits/Diseases

We utilized the LDSC package [13] to compute the genetic correlations between each thyroid hormone (TSH, TT3, FT3, and FT4) and each of the 72 health‐related traits/diseases by inputting the aforementioned GWAS summary statistics along with linkage disequilibrium reference panels developed using HapMap3 [42] variants among European individuals in the 1000 Genomes Project [43].

## 3 Results

**Figure 1:**
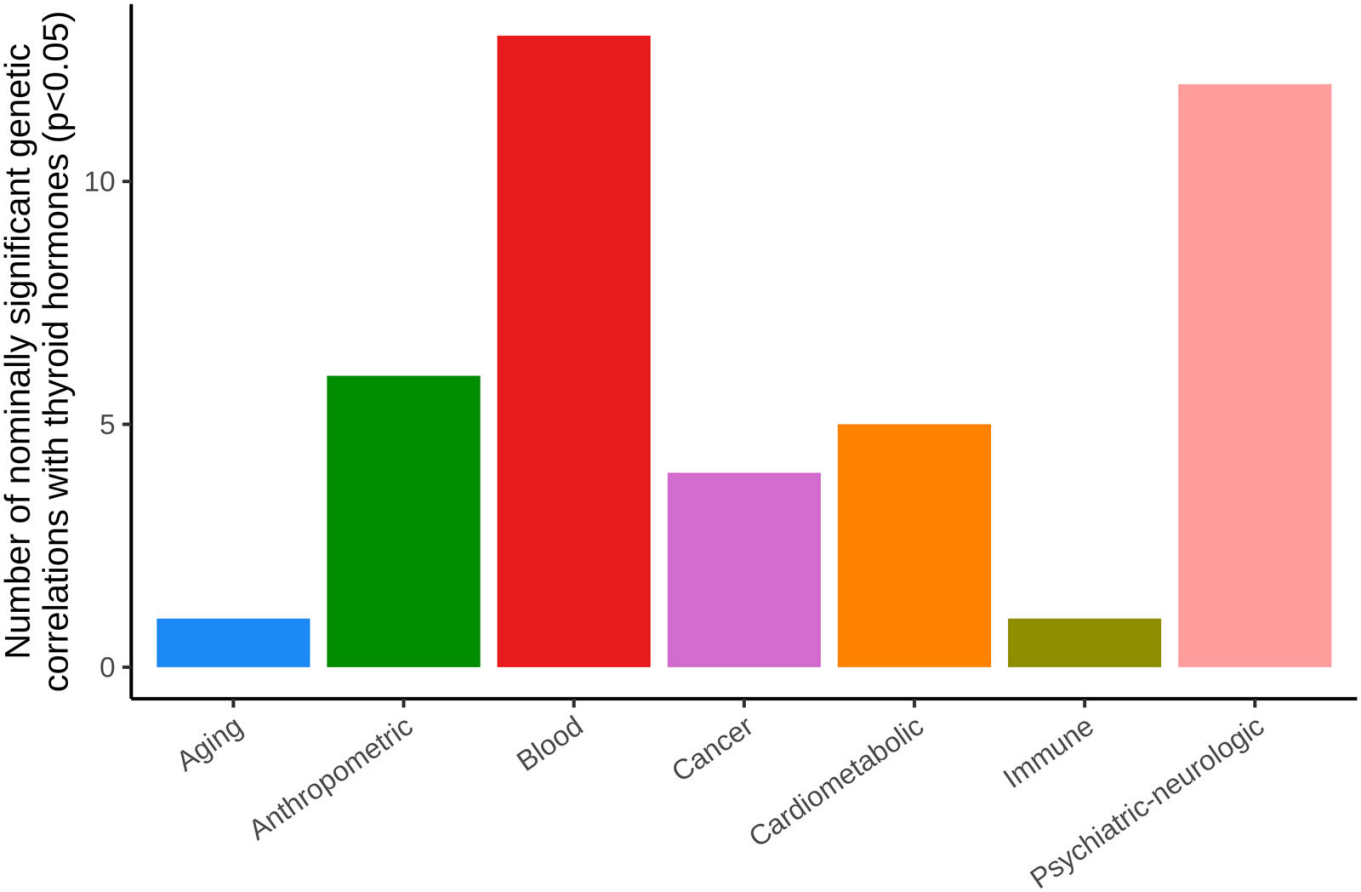
Number of nominally significant genetic correlations with thyroid hormones grouped by categories of health‐related traits and diseases.

We computed the genetic correlations between four thyroid hormones (TSH, TT3, FT3, and FT4) and seventy‐two health‐related traits and diseases using previously published GWAS summary statistics, restricted to variants in the HapMap3 panel. We identified significant genetic correlations between FT3, FT4, and TT3 and seven independent health‐related traits after accounting for multiple testing (FDR‐adjusted *p* < 0.05), including red blood cell counts that exhibited positive genetic correlations with FT3 and FT4 (Table 1); interestingly, TT3 was genetically correlated with body fat percentage, standing height, and depressive symptoms. When relaxing our significance threshold to nominal significance (*p* < 0.05), we identified genetic correlations between all four of the thyroid hormones and 31 unique health‐related traits/diseases (Supporting Information S1: Table 2). At this nominal threshold of significance, several traits/diseases were identified to genetically correlate with more than one thyroid hormone level including overall risk of breast cancer, education years, fasting insulin, fluid intelligence score, hypertension, and granulocyte and myeloid white cell counts (Supporting Information S1: Table 2). Furthermore, when we grouped these health‐related traits/diseases into categories, we observed nominally significant genetic correlations between thyroid hormones and cardiometabolic, blood‐related, immune‐related, and anthropometric traits, as well as psychiatric‐neurologic disorders, cancer, and aging (Figure 1).

**Table 1:** Traits with significant genetic correlations with thyroid hormones after accounting for multiple testing (FDR‐adjusted *p*‐value < 0.05).

Thyroid hormone	Trait name	Genetic correlation	Nominal *p* value	FDR‐adjusted *p* value
Free triiodothyronine (FT3)	Red blood cell count	0.2318 (0.0466)	6.55E‐07	5.11E‐05
	Birth Weight	−0.1941 (0.0579)	8.01E‐04	2.37E‐02
	Reticulocyte count	0.1658 (0.05)	9.13E‐04	2.37E‐02
Free thyroxine (FT4)	Red blood cell count	0.1158 (0.0329)	4.32E‐04	3.37E‐02
Total triiodothyronine (TT3)	Body fat percentage	0.2313 (0.0623)	2.05E‐04	1.23E‐02
	Education years	−0.2766 (0.0768)	3.16E‐04	1.23E‐02
	Depressive Symptoms	0.3477 (0.101)	5.76E‐04	1.50E‐02
	Standing height	−0.1712 (0.0553)	1.96E‐03	3.83E‐02

## 4 Discussion

In this study, we computed genetic correlations between four thyroid hormones (TSH, TT3, FT3, and FT4) and 72 human health‐related traits that were not directly related to thyroid function or disease. In total, we identified seven unique health‐related traits that were genetically correlated with FT3, FT4, and TT3 after correcting for multiple testing, suggesting at least partially shared genetic regulation of thyroid hormone levels and these health‐related traits. We additionally noticed several traits had relatively large, non‐zero genetic correlations with thyroid hormone levels, but were not statistically significant after adjusting for multiple testing; the lack of power in evaluating these genetic correlations may potentially be due to a lack of power in the GWAS summary statistics used to obtain these correlations. Moreover, the genetic correlations computed in this study were restricted to HapMap3 variants, which tend to be a common set of well‐imputed variants across studies, but this restriction may fail to capture genetic correlations that exist across variants outside of the HapMap3 panel.

Among the traits we identified that had significant correlations with levels of thyroid hormones after correcting for multiple testing, we observed red blood cell counts exhibited positive genetic correlations with levels of FT3 and FT4. These results were consistent with previous studies indicating that thyroid hormones may enhance the production of red blood cells by stimulating the production of erythrocyte precursors [44, 45]; additionally, they are consistent with the well‐documented clinical observation of anemia being common among patients with hypothyroidism [44]. Moreover, in vivo experimental studies have demonstrated that thyroxine treatment in mouse models with chronic anemia directly led to erythroblast differentiation and alleviated anemic symptoms through binding to the thyroid hormone receptor β [46]. Furthermore, we noticed FT3 also exhibited positive genetic correlations with abundance of reticulocytes, an immature form of erythrocytes; this observation aligns with previous in vitro work demonstrating that triiodothyronine promotes the proliferation and expansion of immature red blood cells by suppressing the p27^Kip1^ cell cycle inhibitor in fetal liver erythroid progenitor cells [47]. Taken together, these findings suggest that the genetic regulation of thyroid hormone levels may in part be shared with that of erythrocyte abundance.

We also identified several nominally significant genetic correlations including a positive genetic correlation between TSH levels and ER‐positive breast cancer risk and a negative genetic correlation between FT4 levels and ER‐positive breast cancer risk, which both had consistent directionality with previous observational studies [48, 49]. These findings suggest the genetic basis of thyroid hormone levels and breast cancer may be partially shared and warrant additional studies with increased sample sizes. Additionally, previous literature has shown thyroid hormone production is correlated with sleep duration, insomnia and depression [9, 50, 51, 52, 53]. Similarly, in our study, we observed nominally negative genetic correlations between FT4 levels and sleep duration, TT3 levels and sleep duration, and TSH levels and insomnia. We also found nominally positive genetic correlations between TT3 level and depressive symptoms. These findings suggest the presence of shared genetic variants that may pleiotropically influence these traits. Given that insufficient sleep has been found to be associated with more severe depressive symptoms [54, 55], further research is warranted to investigate the potential genetic mechanisms underlying both these traits alongside that of thyroid hormones.

Though this study successfully identified numerous genetic correlations between thyroid hormone metrics and human health‐related traits, it had several limitations. First, the GWAS summary statistics utilized in this study for thyroid hormone metrics were derived exclusively from individuals of European ancestry, as data for thyroid function metrics are currently not as well powered in non‐European populations; hence, additional validation studies of these genetic correlations we identified that are conducted using GWAS summary statistics derived from diverse, non‐European populations are warranted. Furthermore, thyroid hormone levels can vary significantly even within the same individual at different times of the day, such as between morning and evening measurements [56]. Limiting participants in thyroid function GWAS to those from studies with standardized sample collection protocols, including consistent timing for measurements, could enhance the ability to detect genetic correlations with health‐related traits.

In conclusion, while previous clinical and epidemiological studies have observed associations between thyroid hormones and various health‐related traits, this study highlights that the genetic regulation of thyroid hormone levels are in part shared with many of these traits. Further work into examining these genetic correlations utilizing GWAS data derived from non‐European populations, as well as with variants outside of those in the HapMap3 panel, are warranted to further improve our understanding of the shared genetic bases between thyroid hormones and other human health‐related traits.

## Author Contributions

**James L Li:** conceptualization, methodology, formal analysis, project administration, visualization, writing – review and editing, writing – original draft, funding acquisition, investigation. **Yijia Sun:** conceptualization, methodology, formal analysis, visualization, writing – review and editing, writing – original draft, Investigation.

## Conflicts of Interest

The authors declare no conflicts of interest.

## Transparency Statement

The lead author James L. Li affirms that this manuscript is an honest, accurate, and transparent account of the study being reported; that no important aspects of the study have been omitted; and that any discrepancies from the study as planned (and, if relevant, registered) have been explained.

## Supporting information

HSR_SUPPLEMENTARY_TABLES.

## Data Availability

The full summary of genetic correlations between each thyroid hormone and all 72 traits is available at https://github.com/james-li-projects/thyroid_hormone_rG.
